# Steeper or Faster? Tactical Dispositions to Minimize Oxygen Cost in Ski Mountaineering

**DOI:** 10.3389/fspor.2021.828389

**Published:** 2022-01-31

**Authors:** Arnstein Sunde, Fredrik Christoffersen, Jan-Michael Johansen, Øyvind Støren

**Affiliations:** Department of Sports, Physical Education and Outdoor Studies, University of Southeastern Norway, Bø, Norway

**Keywords:** ski mountaineering, work economy, vertical speed, heel elevator, route selection

## Abstract

**Purpose:**

Investigate the effect of speed, inclination, and use of heel elevator on the oxygen cost of vertical climbing (C_vert_) in ski mountaineering.

**Methods:**

In this study, 19 participants who were (3 women and 16 men) moderate- to well-trained recreational Norwegian ski mountaineers were involved. All participants were tested for VO_2max_ in running, and in a ski mountaineering test on a treadmill, to assess C_vert_. The test protocol consisted of 12 4 min work periods at different inclinations from 13 to 23°, with continuous VO_2_ measurements. After every second work period, the inclination increased by 2°, and speed was decreased accordingly. The speed reduction was based on the equation V_vert_ = speed · sin(α), where α represents the angle of inclination. V_vert_ was thus held constant for each work period (854 m·h^−1^). All work periods were completed twice, with and without a heel elevator. Half of the subjects started with the smallest inclination, and the other half started with the steepest inclination.

**Results:**

The results showed that C_vert_ was unchanged at all inclinations except 13°, where there was a significantly higher C_vert_, at the same V_vert_. Only at 13°, C_vert_ was higher with the use of heel elevator. There was also a significant trend indicating lower C_vert_ with use of heel elevator with steeper inclination.

**Conclusions:**

There seemed to be nothing to gain by choosing detours if the inclination was 13° or less. The use of heel elevator was more advantageous, the steeper the inclination, but at 13° there was a negative effect of using heel elevator.

## Introduction

Competitive ski mountaineering involves both uphill and downhill sections. The duration of the competition is relatively long, from 30 to 600 min (Gallaerts et al., 2018). It involves altitude gains of ~80 m in a sprint, up to ~2,000 m in long individual races (Bortolan et al., 2021), with a mean duration of 101 min with an intensity of 93% maximal heart rate (HR_max_) (Duc et al., 2011). Compared to for example cross-country skiers, ski mountaineers are relatively heavy equipped, wearing both rucksack and helmet in addition to ski mountaineering skis and boots. However, skis and boots would not add considerable weight compared to cross country equipment, with a minimum weight of 1 kg per pair of boots and a minimum weight of 1.5 kg per pair of skis in ski mountaineering (Bortolan et al., 2021). Race time has been found to correlate well with maximal oxygen consumption (VO_2max_), indicating ski mountaineering to be a typical aerobic endurance sport (Duc et al., 2011). Also, in ultra-alpine running, aerobic endurance sports in previous studies (Støren et al., 2013, 2014; Sunde et al., 2019; Johansen et al., 2020, 2021) have been shown to depend on the maximal aerobic speed (MAS). As shown in Helgerud et al. (2010) MAS is the product of VO_2max_ divided by the oxygen cost. Consequently, oxygen cost should be considered an important performance determining variable for time performance in the uphill part of ski mountaineering. A lower oxygen cost could cause either a faster or a less demanding way up. This would result in either a shorter time up or a surplus on the way down. In addition to the obvious time gains, a less demanding way up could cause safer route selections according to both avalanche danger and skiing skills in exposed terrain.

Because of the constant change of inclination during ski mountaineering, it should be most appropriate to calculate oxygen cost relative to the vertical displacement (C_vert_), i.e., C_vert_ = ml·kg ^−1^·${\text{m}}_{\text{vert}}^{-1}$. In the uphill parts, C_vert_ is mostly determined by changes in speed, vertical speed (V_vert_), and slope angle (Praz et al., 2016a), while also neuro-muscular efficiency (Barrett-O'Keefe et al., 2012), anthropometrics (Tosi et al., 2010), equipment loads (Tosi et al., 2010), and skiing technique (Skattebo et al., 2019) are important contributors.

Previous studies have found C_vert_ not to be affected by the speed at low inclinations (Praz et al., 2016a,b,a,b). At higher inclinations, C_vert_ has been found to be lowest at 3.3 km·h^−1^ and increasing at both lower and higher speeds (Tosi et al., 2010; Praz et al., 2016a,b). However, previous studies have not measured C_vert_ over a broad range of inclinations, including steep inclinations above 20°. Also, previous studies have been conducted on snow or roller skies on a treadmill (Tosi et al., 2009, 2010; Duc et al., 2011; Praz et al., 2016a,b; Gallaerts et al., 2018). No previous studies have used ski mountaineering equipment on a treadmill.

To some extent, ski mountaineering gives the participants the possibility to choose their own route both uphill and downhill. This implies that they can make a steep but shorter track, or a less steep zigzag patterned track, resulting in a long way to the top. In addition, the skiers must consider when to raise their heel elevators when the tracks get steeper. However, in competitions, bindings are fixed in height. The use of heel elevators is thus most interesting in recreational ski mountaineering. When using heel elevators, the boots will be positioned more horizontally during the propulsive phase, which is meant to give a less strenuous way up for the skier. This means that there are some tactical considerations that may affect overall performance. No previous studies have investigated the effect of heel elevators on C_vert_ at different inclinations.

Analyzes of C_vert_ with different inclinations, different speeds, and consequently a choice of using a heel elevator, are thus of special interest in ski mountaineering. While speed and inclinations are important considerations in ski mountaineering both in competitions and as a leisure activity, the use of heel elevators will only be interesting in the leisure part of ski mountaineering. Therefore, the present study analyzed ski mountaineering in the lab, using ski mountaineering skies and boots on a treadmill at inclinations from 13 to 23°. The present study was also investigating at what inclinations C_vert_ would be the lowest, given the same V_vert_, and at what inclinations the use of heel elevator would give the lowest C_vert_. The hypotheses were that change of inclination would affect C_vert_ at a constant V_vert_ and that the use of heel elevator would improve C_vert_ at the steepest inclinations.

## Methods

### Subjects

The study involved 19 (3 women and 16 men) moderate- to well-trained 32.6 ± 11.1-year-old recreational Norwegian ski mountaineers who participated in this study (Table 1). No measurements of C_vert_ with ski mountaineering equipment had previously been done prior to this study. Based on measurements in mechanical efficiency, by Praz et al. (2016a), on roller skies at low vs. medium inclinations, we calculated the sample size. Given a mean difference in the mechanical efficiency of 13% between inclinations of 10 and 14°, a sample size of 16 participants would be needed. Nineteen participants were recruited to account for possible withdrawals. The study was approved by the institutional research board at the University of Southeastern Norway, the Norwegian Centre for Research Data (NSD), and conducted in accordance with the Helsinki declaration. All subjects gave their written consent to participate, after having received information about the study.

**Table 1:** Characteristics of skiers.

	**All (*N* = 19)**	**Females (*N* = 3)**	**Males (*N* = 16)**
BW (kg)	75.9 ± 10.5	59.5 ± 5.7	79.9 ± 7.2
Age (yrs)	32.6 ± 11.1	35.3 ± 3.5	32.7 ± 12.2
VO_2max_ (mL·kg^−1^·min^−1^)	57.2 ± 6.1	54.3 ± 5.8	58 ± 6.3

*Values are mean ± standard deviation. BW, body weight; VO_2max_, maximal oxygen uptake in running; mL·kg^−1^·min^−1^, milliliters oxygen per kilogram body weight per minute*.

### Test Procedures

The subjects were tested over two different days. Day one consisted of an incremental running VO_2max_ test. VO_2max_ was tested partly to obtain information on the participants' aerobic capacity *per se*, and also to investigate if VO_2max_ could have any impact on C_vert_. The subjects started at an intensity of 8–12 km·h^−1^ and a 5.3% inclination. Every 30 s the speed was increased by.5 km·h^−1^. The test terminated at voluntary fatigue, and additionally heart rate (HR) ≥ 98 % of HR_max_, respiratory exchange ratio (RER) ≥ 1.05, as well as a plateau of the VO_2_ curve, was used to evaluate if VO_2max_ was obtained (Åstrand et al., 2003). The mean of the three subsequent highest registered VO_2_-values, each representing 10 s intervals by the mixing chamber, was set as VO_2max_. All VO_2_ measurements were made by the metabolic test system, Metalyzer II Cortex (Biophysic GmbH, Leipzig, Germany), with a mixing chamber. The treadmill used for running was a Woodway PPS 55 sport (Waukesha, USA). All HR measurements were made by Polar s610 HR monitors (Kempele, Finland).

The second day of testing consisted of a ski mountaineering test on a cross-country skiing treadmill (Rodby RL 2700E, Rodby Innovation, Vänge, Sweden). Ski mountaineering equipment, including skis, boots, poles, and a rucksack of 4 kg, was used on the treadmill. The subjects were acquainted with the cross-country skiing treadmill by use of a 10-min submaximal workout ahead of the test. The test protocol consisted of twelve 4 min work periods with maximal 30 s breaks in between. All work periods were completed twice, with the heel elevator lifted (6.1 cm) every second work period. After every second work period, speed was decreased and inclination increased, or vice versa. This resulted in the same V_vert_ for all periods (Table 2). V_vert_ was calculated from the trigonometric function V_vert_ = speed · sin(α), were α represents the angle of inclination.

**Table 2:** Physiological results from sub-maximal workloads (*N* = 19).

**Work period**	**Inclination (°)**	**V (km·h^**−1**^)**	**Heel elevator (cm)**	**HR (b·min^**−1**^)**	**CF (s·min^**−1**^)**	**VO_**2**_ (mL·kg^**−1**^·min^**−1**^)**	**RPE**	**C_**vert**_ (mL·kg^**−1**^·${\text{m}}_{\text{vert}}^{-1}$)^§^**
1	13	3.8	0.0	167 ± 15	82 ± 7	44.7 ± 3.3	15 ± 3	3.14 ± 0.23^*^
2			6.1	170 ± 14	85 ± 8	46.6 ± 2.8	15 ± 3	3.28 ± 0.20^*^^#^
3	15	3.2	0.0	168 ± 15	72 ± 8	43.2 ± 2.9	14 ± 2	3.03 ± 0.21
4			6.1	169 ± 15	77 ± 7	42.8 ± 2.8	14 ± 3	3.00 ± 0.20
5	17	2.9	0.0	167 ± 16	68 ± 6	42.4 ± 2.6	14 ± 2	2.98 ± 0.18
6			6.1	168 ± 16	71 ± 6	42.1 ± 2.3	14 ± 2	2.96 ± 0.16
7	19	2.6	0.0	167 ± 16	65 ± 6	42.6 ± 2.3	14 ± 2	2.99 ± 0.16
8			6.1	167 ± 16	66 ± 9	41.6 ± 2.0	14 ± 2	2.92 ± 0.14
9	21	2.4	0.0	167 ± 17	63 ± 6	42.5 ± 1.9	15 ± 2	2.98 ± 0.14
10			6.1	167 ± 16	65 ± 5	41.4 ± 2.1	14 ± 2	2.91 ± 0.15
11	23	2.2	0.0	165 ± 18	61 ± 5	43.0 ± 2.1	15 ± 2	3.02 ± 0.15
12			6.1	165 ± 18	63 ± 7	41.5 ± 2.3	14 ± 2	2.91 ± 0.16

*Vertical velocity (Vvert measured as mvert· h^−1^), was held constant at 854 mvert· h^−1^ in all work periods. Values are mean ± standard deviation (SD). °, degrees; V, speed; km·h^−1^, kilometer per hour; HF, heart frequency; b·min^−1^, beats per minute; CF, cycle frequency; s·min^−1^, steps per minute; VO_2_, oxygen uptake; mL·kg^−1^·min^−1^, milliliters per kilogram bodyweight per minute; RPE, rate of perceived exertion measured by Borg scale; C_vert_, oxygen cost of vertical displacement; mL·kg^−1^ ·mvert^−1^, milliliter oxygen per kg bodyweight per vertical meter*.

* *p < 0.01: higher than in all other inclinations*.

# *p < 0.01: worse effect of heel elevator than in all other inclinations*.

§ *p < 0.01: lower C_vert_ with increased inclination*.

Vertical speed (V_vert_) was set to correspond to a relatively fast ski mountaineering speed (854 m_vert_ h^−1^ equivalent to 14.2 m_vert_ · min ^−1^). Oxygen consumption, HR, and cycle frequency (CF) were measured continuously during all 12 work periods. Borg scale was registered immediately after completing each work period. All tests at test day 2 were performed with the Jaeger Vyntus CPX with a mixing chamber, measuring every 20 s (CareFusion, GmbH, Hoechberg, Germany).

The duration of the test was ~55 min. In order to evaluate if changes in HR, VO_2_, and Borg scale was a result of the long duration of the test, or a result of the change of speed and inclination, half of the subjects started with the smallest inclination (work period 1, 2, 3, etc.), and 50% started with the steepest inclination (work period 12, 11, 10, etc.).

### Data Processing and Statistical Analyzes

Oxygen consumption and HR was calculated as the average of three measurements in minute 3–4 (3:00, 3:20, and 3:40). VO_2max_ was set as the mean of the three subsequent highest registered VO_2_-values. CF was registered manually by counting the number of strides from minute 2:00 to 2:30 and multiplied by two in order to get stride · min^−1^.

Normality was tested by the use of QQ-plots and Shapiro-Wilk (*p* = 0.313) and found to represent normal distributions for the main variable (C_vert_). Values were thus expressed descriptively as mean ±*SD*. Correlations between C_vert_, CF and VO_2max_ were expressed as the correlation factor *r* from Pearson's bivariate tests. Based on the correlation coefficient definitions by Hopkins (2000), we have defined strong correlations to be *r* > 0.7 in the present study. Also, in order to detect possible patterns between inclination and work economy, and between velocity and work economy, a general linear model (GLM) with Tukey *post-hoc* tests was performed. Statistical analyzes were performed using the software program statistical package for social science version 26 (SPSS, IBM, Chicago, IL, USA). A *p* < 0.05 was accepted as statistically significant in all tests.

## Results

### The Effects of Speed and Inclinations on C_vert_

All results are presented on a group level in Table 2. There was no significant change (*p* = 0.1) in C_vert_ with increasing inclination when not correcting for the use of heel elevator. However, when corrected for heel elevation there was a significant improvement in C_vert_ with increased elevation (*p* = 0.02). When comparing the work period at 13° with all the other inclinations, there was a significant (*p* < 0.01) higher C_vert_ at 13°.

### The Effect of Heel Elevation on C_vert_

**Figure 1:**
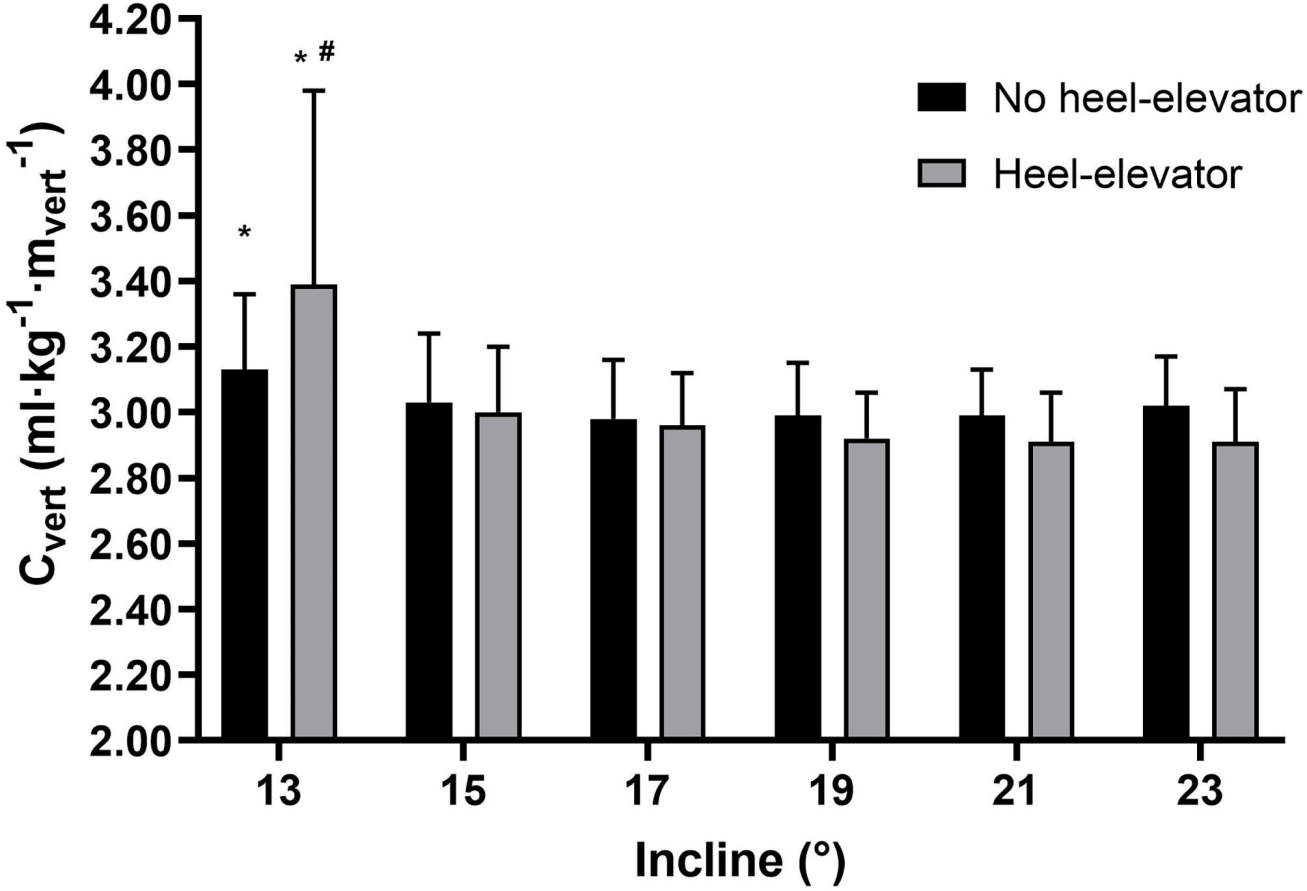
Cost of vertical climbing (C_vert_) for all 12 work periods with and without heel elevator divided into a total of six different inclinations. C_vert_, cost of ski mountaineering; ml kg^−1^ ${\text{m}}_{\text{vert}}^{-1}$, milliliter oxygen per kg bodyweight per minute per vertical meter, incline; °, degrees. *p < 0.01: higher than in all other inclinations. #*p* < 0.01: worse effect of heel elevator than in all other inclinations.

When comparing differences in C_vert_ related to the use of heel elevators, there was a significant trend (*p* < 0.01) using heel elevators with increasing inclination. At 13° the *post hoc* analyzes showed that C_vert_ was significantly (*p* < 0.01) worse with the use of the heel elevator compared to all other inclinations. The results for C_vert_ with or without heel elevators are presented in Figure 1.

### Associations Between Cycle Frequency, Rate of Perceived Exertion, Heart Rate, and C_vert_

CF decreased significantly (*p* < 0.01) with an increase in inclination and decrease in speed. However, there was no correlation (*r* = 0.27) between CF and C_vert_. There were no correlations at any inclinations between running VO_2max_ and C_vert_ or between running VO_2max_ and CF.

There was no change in RPE or HR throughout the 12 work periods for the whole group.

## Discussion

### Main Findings

The main findings of the present study were that C_vert_ was unchanged at all inclinations except 13°, where there was a significantly higher C_vert_ when tested with the same V_vert_ (854 m·h^−1^). When corrected for heel elevator there was a significant trend between increased inclination and a decrease in C_vert_. The use of heel elevator was more advantageous, the steeper the inclination with no positive effect at 13°.

### The Effects of Speed and Inclinations on C_vert_

Typical speed during guided ski mountaineering as a leisure activity is normally ~400 and 600 m_vert_ · h^−1^ when guiding experienced groups. During competing ski mountaineering, the V_vert_ could differ from ~550 to 900 m_vert_ · h^−1^ (Tosi et al., 2009; Duc et al., 2011; Praz et al., 2014). This means that the chosen V_vert_ in the present study (854 m_vert_ · h^−1^) corresponds to competitive ski mountaineering and not ski mountaineering as a leisure activity. To keep the chosen V_vert_ in this study, the speed at 13° had to be high (3.8 km · h^−1^), probably higher than most of the participants were familiar with. When the inclination decreases, speed must increase in order to maintain the same V_vert_. The increase in V per decrease in degrees is exponential. Because of this, speed was unproportionally high at 13° and this may have caused the high C_vert_ at this inclination. This V_vert_ is equivalent to 4.1 km · h^−1^ at 11.9°. The chosen V_vert_ in the present study thus represents a higher speed than the optimal speed of 3.3 km · h^−1^ at 11.9°, presented in Tosi et al. (2010). Comparably, the same V_vert_ as in the present study would have resulted in a speed of 8.6 km · h^−1^ at 5.7° in the study by Praz et al. (2016a). However, the highest speed at 5.7° in Praz et al. (2016a), was 6 km · h^−1^. Although the principles in this comparison may be valid, caution should be taken regarding the actual speeds, as both Tosi et al. (2010) and Praz et al. (2016a) used rollerskis. In other words, there seemed to be some sort of threshold between 13 and 15° where increased speed would lead to impaired C_vert_. The present results should be considered specifically for ski mountaineering. In, e.g., alpine running, Savoldelli et al. (2017) found a more continuous reduction of Cvert the steeper the inclination without a marked threshold.

Praz et al. (2016a,b) did not find any changes in C_vert_ with changes in speed up to ~6° but found that C_vert_ decreases with increasing speed at steeper inclinations. The present study did not investigate different speeds at the same inclinations, but rather different inclinations at the same V_vert_. This makes direct comparisons somewhat difficult.

### The Effect of Heel Elevation on C_vert_

The present results displayed a larger gain from using heel elevator the higher the inclination. At 13° the effect of heel elevator was negative and significantly worse than at all other inclinations. These results provide novel data on the use of heel elevators at different inclinations, and we suggest not to use heel elevators at 13° or less inclination. No previous studies have investigated the effects of heel elevators on C_vert_ at different inclinations. Comparisons with previous studies are therefore difficult.

### Cycle Frequency and C_vert_

Mean CF in the present study ranged from 61 to 85 strides · min^−1^, increasing with increasing speed. 85 strides · min^−1^ was thus obtained at 3.8 km · h^−1^. In Praz et al. (2016a) mean CF ranged from 29 to 47 strides · min^−1^, with 47 strides · min^−1^ at 6 km · h^−1^. However, the skiers in Praz et al. (2016a) were using roller skies, probably explaining the lower CF. In a recent study by Lasshofer et al. (2021), CF was measured during an incremental test at 14°. Mean CF in that study was 97 strides · min^−1^, with 137 strides · min^−1^ at the highest speed of 6.9 km · h^−1^. These results taken together may indicate that CF increases with increasing speed when using ski mountaineering equipment as opposed to roller skis on a treadmill. In the present study, CF decreased significantly with an increase in inclination and decrease in speed, but there was no correlation (*r* = 0.27) between CF and C_vert_. There were also no correlations at any inclinations between the subject's condition measured as VO_2max_ in running and CF. The participants in the present study may be characterized as a homogenous group regarding ski mountaineering experience. It seems that they subconsciously chose the most effective CF based on their previous experiences. Tosi et al. (2010) suggested that higher CF resulted in a higher total work at a given speed. In opposition to the results in Tosi et al. (2010), there were relatively small variations in CF in the present study, expressed by coefficients of variance (CV) between 8 and 13% in the 12 work periods.

### Limitations and Practical Implications

At the chosen speed it looks like route selection does not matter relative to C_vert_ given inclinations between 15 and 23°. It must be emphasized that 854 m·h^−1^ is a relatively fast competition speed that requires a good shape to maintain over a long time. It is conceivable that testing at lower speeds could have given different results.

The present study showed a significant trend indicating lower C_vert_ with the use of heel elevators with steeper inclination. There are nevertheless individual differences. As shown in Table 2, quite a few of the participants are profiting at 17°, and some are already at 15°. It may therefore be appropriate on an individual basis, to test both with and without heel elevation at inclinations between 15 and 19°, to evaluate what is perceived as most effective.

In running and cycling, Støren et al. (2008) and Sunde et al., 2010, respectively, discussed that improvement in oxygen cost corresponded to an equivalent better time performance. The difference between C_vert_ at 13° and the mean C_vert_ in all the other inclinations was 6.5%. A 6.5% improvement in C_vert_, could therefore correspond to a 6.5% improvement in ascent time performance in a ski mountaineering competition. Duc et al. (2011) showed that during a ski mountaineering race with two uphill sections and a total duration of 95, 11 min was descents and 44 ascents. This means that a 6.5% improvement in C_vert_ could provide an improved overall time performance of ~3 min.

### Future Perspectives

Future studies should analyze time performance both in the lab and in the field, to detect correlations between vertical MAS, C_vert_, VO_2max_, and time performance in the uphill sections of ski mountaineering. Also, the relevance and strong validity of field testing are valuable despite the challenges of the variations in the snow- and weather conditions, steepness and sloping terrain in test tracks, change of use of muscles at each turn, and so forth. Laboratory-based tests will never be 100% equal to ski mountaineering at snow but have the advantage of being able to standardize and ensure equal conditions from test to test.

Considering this study found that the use of heel elevator was more advantageous the steeper the inclination, it would be interesting to analyze how the use of heel elevator affects both the way the muscles work and biomechanical factors.

It could also be interesting to analyze how changes in e.g., maximal leg strength affects C_vert_. Previous studies have revealed improvements of ~5% in oxygen cost after maximal strength training in both cross country skiing (Østerås et al., 2002), running (Støren et al., 2008), and cycling (Sunde et al., 2010). In these studies, a decrease in oxygen cost led to a corresponding improvement in time performance. This may mean that if ski mountaineers add a relatively small amount of maximal strength training in, e.g., half squat, it could lead to better performance. Controlled interventions targeting adaptations in VO_2max_, and the consequent impact on-time performance and C_vert_ would also be of great interest. The suggested future investigations would also benefit from investigating potential sex differences in tactical dispositions related to C_vert_. Also, the incorporation of accelerometers to determine the energy expenditure during ski mountaineering could further add help to the tactical dispositions. To better understand this topic, it is suggested to read (e.g., Crouter et al., 2006; Kinnunen et al., 2019; Afaq et al., 2020).

## Conclusions

C_vert_ was unchanged at all inclinations except 13°, where there was a significantly higher C_vert_ when tested with the same V_vert_ (854 m·h^−1^). When corrected for heel elevator there was a significant trend between increased inclination and a decrease in C_vert_. The use of heel elevator was more advantageous, the steeper the inclination with a negative effect at 13°.

Therefore, it seemed to be nothing to gain by choosing detours or heel elevator if the inclination is 13° or less.

## Data Availability Statement

The original contributions presented in the study are included in the article/supplementary material, further inquiries can be directed to the corresponding author.

## Ethics Statement

The studies involving human participants were reviewed and approved by the Institutional Research Board at the University of Southeastern Norway and the Norwegian Centre for Research Data (NSD). The patients/participants provided their written informed consent to participate in this study.

## Author Contributions

AS: planning, testing, and writing. FC: testing and contributed to the writing. J-MJ: statistical analyzes and contributed to the writing. ØS: supervisor and contributed to the writing. All authors contributed to the article and approved the submitted version.

## Conflict of Interest

The authors declare that the research was conducted in the absence of any commercial or financial relationships that could be construed as a potential conflict of interest.

## Publisher's Note

All claims expressed in this article are solely those of the authors and do not necessarily represent those of their affiliated organizations, or those of the publisher, the editors and the reviewers. Any product that may be evaluated in this article, or claim that may be made by its manufacturer, is not guaranteed or endorsed by the publisher.
